# Phylogenetic Diversity, Antimicrobial Susceptibility and Virulence Characteristics of *Escherichia coli* Isolates from Pigeon Meat

**DOI:** 10.3390/antibiotics8040259

**Published:** 2019-12-10

**Authors:** Rosa Capita, Jorge Cordero, Diana Molina-González, Gilberto Igrejas, Patrícia Poeta, Carlos Alonso-Calleja

**Affiliations:** 1 Department of Food Hygiene and Technology, Veterinary Faculty, University of León, 24071 León, Spain; 2 Institute of Food Science and Technology, University of León, 24071 León, Spain; 3 Associated Laboratory for Green Chemistry, University NOVA of Lisboa, 2829-516 Caparica, Portugal; 4 Department of Genetics and Biotechnology, University of Trás-os-Montes and Alto Douro, 5000-811 Vila Real, Portugal; 5 Functional Genomics and Proteomics Unit, University of Trás-os-Montes and Alto Douro, 5000-811 Vila Real, Portugal; 6 Department of Veterinary Sciences, University of Trás-os-Montes and Alto Douro, 5000-811 Vila Real, Portugal

**Keywords:** *Escherichia coli*, antibiotic resistance, virulence factors, phylogenetic groups, pigeon meat

## Abstract

Monitoring resistance to antibiotics in wild animals may assist in evaluating tendencies in the evolution of this major public health problem. The aims of this research work were to determine the patterns of antibiotic resistance in *Escherichia coli* isolates from the meat of wild or domestically reared pigeons from Spain, to detect the presence of virulence and antibiotic resistance genes, and to carry out a phylogenetic classification of the isolates. Of the 37 *E. coli* strains tested, 32.43% of them belonged to the B2 phylogenetic group, which is often implicated in extra-intestinal infections. None of the strains showed extended-spectrum beta-lactamase activity. All the isolates presented resistance or reduced susceptibility to two or more antibiotics, with high levels of resistance to β-lactams, aminoglycosides and tetracycline. Ten resistance genes were detected, the most frequent of which were *amp*C, conferring resistance to ampicillin and *aad*A, conferring resistance to streptomycin. In total, 97.30% of the strains carried virulence factors (between one and five). The strains from pigeons reared in captivity harboured higher average numbers of resistance and virulence genes than isolates from wild pigeons. Pigeon meat is an important reservoir of *E. coli* with genes for antibiotic resistance and virulence having the potential to cause disease in humans.

## 1. Introduction

*Escherichia coli* is a common bacterium in the gut of humans and animals. Most strains of *E. coli* are not pathogenic to humans and are seen solely as indicators of faecal contamination. However, between 10% and 15% of strains are pathogenic and able to cause a wide range of illnesses, mainly due to the consumption of contaminated food or drink [1,2]. To this effect, *E. coli* was responsible for 0.9% (48 out of a total of 5,079) of the outbreaks of foodborne diseases in the European Union in 2017 [3]. This microorganism is also a cause for concern in the medical field, where it is responsible for a considerable percentage of nosocomial infections [4]. 

Resistance to antibiotics has been defined as one of the greatest threats to public health and one of the main challenges in medicine for the twenty-first century [5]. *E. coli* has a noteworthy capacity to acquire antibiotic resistance genes as a result of the efficient horizontal transfer mechanisms these microorganisms have developed over time [6]. Hence, strains of this bacterial group act as reservoirs of resistance genes. This is a worrying fact in the context of public health, since there is a high likelihood of transfer of genes to other, pathogenic, bacteria. It also allows this bacterial group to be used as sentinel for resistance to antibiotics [6].

The acquisition of antibiotic resistant bacteria by wild animals contributes to increasing the problem of resistance, given that these animals constitute an environmental reservoir of genes and are a biological mechanism for spreading them into the general surroundings [7]. This circumstance enables these animals to be used as environmental indicators of resistance to antibiotics. Moreover, in species used for human consumption, as is the case with pigeons, there is a further problem of potential infection of consumers by resistant bacteria as an outcome of cross-contamination or insufficiently cooked foodstuffs [8].

In recent years, several studies have reported increasing resistance in bacteria isolated from wild animals [9,10,11]. This circumstance makes it feasible to use these animals as indicators of resistance to antibiotics in the environment. However, there is very limited research on phenotypic and genotypic characterization of *E. coli* isolates from pigeons in Spain. The aims of this research work were to determine the patterns of antibiotic resistance among a collection of *E. coli* strains isolated from the meat of pigeons from north-west Spain for the purpose of performing a phylogenetic classification of the strains and detecting the genes for resistance to antibiotics and the virulence factors present. A comparison was made of the findings for isolates from both wild pigeons and others raised in captivity. 

## 2. Results

### 2.1. Antibiotic Susceptibility

The *E. coli* strains showed resistance or reduced susceptibility to two (2.70% of strains), three (13.51%), four (40.54%), five (27.02%), six (5.41%), seven (2.70%) and even eight (8.11%) antibiotics. Considering resistance in the strict sense only (excluding strains with reduced susceptibility), 40.54% of the strains were susceptible, while 40.54% showed resistance to one antibiotic, and 18.92% showed resistance to two antibiotics. The average number of resistances per strain was 0.78 (resistance in the strict sense only) or 4.59 (resistance together with reduced susceptibility).

**Figure 1:**
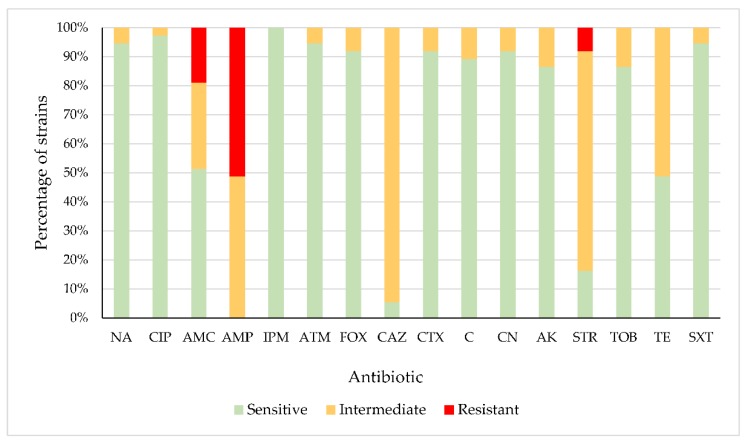
Percentage of *Escherichia coli* strains sensitive, intermediate or resistant to each antibiotic tested. Nalidixic acid (NA), ciprofloxacin (CIP), amoxicillin-clavulanic acid (AMC), ampicillin (AMP), imipenem (IPM), aztreonam (ATM), cefoxitin (FOX), ceftazidime (CAZ), cefotaxime (CTX), chloramphenicol (C), gentamicin (CN), amikacin (AK), streptomycin (STR), tobramycin (TOB), tetracycline (TE) and sulfamethoxazole- trimethoprim (SXT).

None of the strains were extended-spectrum beta-lactamase producers. Figure 1 shows the percentages of strains that were sensitive, intermediate (with reduced susceptibility) or resistant to each of the antibiotics tested. The strains presented a high prevalence of resistance or reduced susceptibility to β-lactams, aminoglycosides and tetracycline. There were percentages of strains with resistance or reduced susceptibility amounting to 48.65% (amoxicillin-clavulanic acid), 100% (ampicillin), 94.59% (ceftazidime), 83.78% (streptomycin) and 51.35% (tetracycline). For the remaining antibiotics, the percentages of strains showing resistance or reduced susceptibility ranged between 2.70% and 13.51%, the exception being imipenem, to which all strains were sensitive. 

A greater prevalence of resistant strains (*P* < 0.05) was observed among strains from birds reared in captivity (70.83% of these presented resistance to at least one antibiotic). In contrast, wild birds showed less prevalence (38.46% had resistance to at least one antibiotic). 

Table 1 shows the various patterns of resistance found and the number of strains associated with each. Four patterns stood out as particularly frequent, amoxicillin-clavulanic acid/ampicillin/ceftazicime/streptomycin/tetracycline (shown by seven strains; 18.92% of the total), amoxicillin-clavulanic acid/ampicillin/ceftazidime/streptomycin (six strains; 16.22%), ampicillin/ceftazidime/streptomycin (three strains; 8.11%) and ampicillin/ceftazidime/streptmycin/tetracycline (three strains; 8.11%).

### 2.2. Genotypic Characterization

A total of 28 genes were studied. Three of them served to ascribe the strains to phylogenetic groups, 18 were antibiotic resistance genes and seven were genes encoding virulence determinants. The classification into phylogenetic groups placed the strains in Groups A (two strains; 5.41% of the total), B1 (23 strains; 62.16%) and B2 (12 strains; 32.43%). No strain from Group D was detected. No significant differences were observed between groups of strains (from wild or domestic birds) regarding their ascription to the various phylogenetic groups.

**Figure 2:**
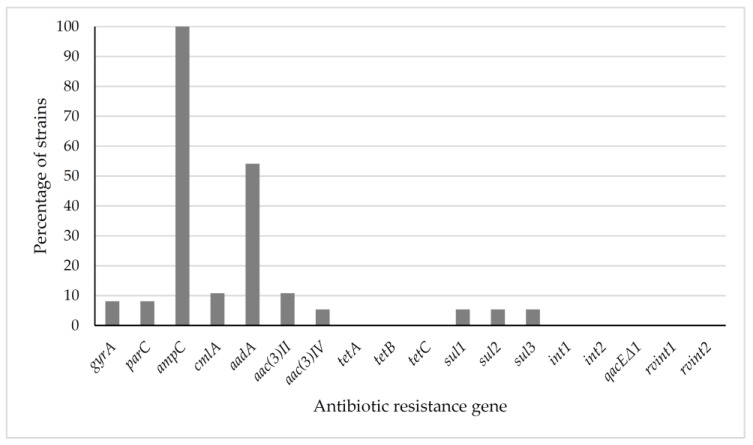
Percentage of *Escherichia coli* strains harbouring each antibiotic resistance gene studied. Genes for resistance to quinolones (*gyr*A, *par*C), β-lactams (*amp*C), chloramphenicol (*cml*A), streptomycin (*aad*A), gentamicin (*aac*(3)II, *aac*(3)IV), tetracycine (*tet*A, *tet*B, *tet*C) and trimethoprim-sulfamethoxazole (*sul*1, *sul*2, *sul*3) were studied by PCR. The resence of the *intl*1 and *intl*2 genes, encoding Class 1 and 2 integrases, respectively, their variable region (*vrintl*1, *vrintl*2), and the presence of the *qac*EΔ1 region, were also studied.

Figure 2 shows the percentage of strains (relative to the 37 strains studied) that carried each of the genes conferring resistance to the antibiotics analysed. Overall, 79 genes giving resistance to antibiotics were detected in the 37 isolates investigated. The strains from pigeons reared in captivity presented a greater (*P* < 0.05) prevalence of resistance genes (2.42 genes per strain) than those from wild pigeons (1.62 genes per strain). Similar average numbers of resistance genes were observed in the strains belonging to the different phylogenetic groups.

The three strains that were resistant or intermediate to quinolones and fluoroquinolones (nalidixic acid or ciprofloxacin), representing 8.11% of all the strains of *E. coli* tested, had the *gyr*A and *par*C genes. The *amp*C gene was studied in all the strains (all the isolates showed resistance or reduced susceptibility to ampicillin) and was detected in 100% of them.

The *cml*A gene was present in the four strains presenting resistance or reduced susceptibility to chloramphenicol (10.81% of all the strains). Furthermore, the *aad*A, *aac*(3)II and *aac*(3)IV genes, which were analysed in the 32 strains with resistance or reduced susceptibility to aminoglycosides, were found in 20 strains (*aad*A), four strains (*aac*(3)II) and two strains (*aac*(3)IV), respectively.

No genes for resistance to tetracycline (*tet*A, *tet*B and *tet*C) were detected in any of the 19 strains tested. Last, the *sul*1, *sul*2, *sul*3, *intl*1, *intl*2, *qac*EΔ1, *rvintl*1 and *rvintl*2 genes were analysed in the two strains presenting resistance or reduced susceptibility to SXT. The *intl*1, *intl*2, *rvint*1, *rvint*2 and *qac*EΔ1 genes were not found in any of the strains checked, while the *sul*1, *sul*2 and *sul*3 genes were detected, all of them in both strains (5.41% of the total).

Several genes for virulence were detected: *aer* (21 strains; 16 from phylogenetic group B1 and five from phylogenetic group B2), *cnf*1 (eight strains; five B1 and three B2), *fim*A (35 strains; 23 B1 and 12 B2), *hly* (16 strains; four B1 and 12 B2) and *pap*GIII (nine strains; one A, and eight B2). The *pap*C and *stx*_1_ genes were not detected. Table 2 shows a grouping of strains as a function of the number of virulence factors they bore. Several virulence patterns stand out: *aer/fim*A (14 strains; 37.84%), *aer/fim*A*/hly/pap*GIII (five strains; 13.51%), *fim*A*/hly* (four strains; 10.81%) and *cnf1/fim*A*/hly* (four strains; 10.81%). Of the 89 virulence factors encountered in the overall set of strains trialled, 72 were detected in strains from birds raised in captivity (3.00 virulence factors per strain), and 17 in isolates from wild pigeons (1.31 virulence factors per strain; *P* < 0.05). The strains from phylogenetic groups B1 and B2 harboured similar (*P* > 0.05) average numbers of virulence factors (48 and 40, respectively).

## 3. Discussion

### 3.1. Antibiotic Susceptibility

In recent years, *E. coli* isolates producing extended-spectrum β-lactamases (ESBLs) have become a serious health problem worldwide [12]. In the study presented here, the double disc synergy test for detecting ESBLs was negative for all the *E. coli* isolates. The absence of ESBLs is in line with the results of other researchers who have investigated both domestic and wild birds [9,13,14,15]. 

The considerable prevalence of resistant or intermediate strains observed in this research work is a worrying finding considering that antibiotic resistance undermines the usefulness of these compounds in clinical practice. Along these lines, infections caused by multi-resistant bacteria are associated with high morbidity and mortality rates, in addition to increased treatment costs [16,17]. Notably, over 50% of the strains showed resistance or reduced susceptibility to ampicillin, ceftazidime, streptomycin and tetracycline, which are categorized as “critically important antimicrobials” (ampicillin, ceftazidime and streptomycin) or “highly important antimicrobials” (tetracycline) for human medicine [18]. According to the World Organization for Animal Health [19], ampicillin, streptomycin and tetracycline are classified as “critically important antimicrobial agents” in veterinary medicine. High levels of resistance to these antimicrobials have also been reported among various animal species, including pigeons [9,10,11,12,13,20,21,22,23,24,25]. 

The high prevalence of strains showing resistance or reduced susceptibility found in this study coincides with the outcomes of other research work undertaken in north-west Spain focused on chickens [26,27], game [28] and meat preparations [29,30]. Specifically regarding pigeons, a high prevalence of resistance has been noted in Spain [31], Japan [20], Belgium [13], Brazil [32], the Czech Republic [9], Poland [25], Bangladesh [33] and Slovakia [12]. Other researchers have also detected a considerable presence of resistance to antibiotics in strains of *E. coli* derived from the droppings of various kinds of wild birds and mammals living in close proximity to humans [9,24,34,35].

The results obtained in this research work highlight an association between the level of antimicrobial resistance in *E. coli* isolates from pigeon meat and these birds’ proximity to human populations. To this effect, the highest rates of resistance to antibiotics were found in strains isolated from animals kept in captivity. Similar findings have been reported by other authors [36]. Notably, exposure to anthropogenic factors such as human refuse or livestock farming may encourage resistant bacteria to colonise these birds, while also facilitating exposure to antimicrobial medication, antimicrobial residues or resistant genes, contributing to the development and maintenance of antibiotic resistance in the microbiota of these animals [6,10,37,38]. In fact, several antibiotics to which considerable prevalence of resistance was observed (e.g., tetracycline) are regularly used as prophylactic or therapeutic agents in animal production [39]. It should be noted, however, that due to the impact of both livestock and human densities on the environment, very slight differences between domestic and wild pigeons with respect to resistance to antibiotics were reported in a previous study [31].

### 3.2. Genotypic Characterization

*Escherichia coli* is classified into various phylogenetic groups, which differ in their ecological niches, characteristics and capacity to cause diseases. Isolates belonging to phylogenetic Groups A and B1, to which most of the strains in this research were ascribed, are commensals and only exceptionally associated with human extra-intestinal infections. In such cases, this is a consequence of the horizontal transfer of genes coding for virulence [38]. The predominance of strains from these phylogenetic groups is a finding that coincides with the results obtained by other authors on examining strains from different animal species [11,15,40,41,42,43]. In contrast, Groups B2 and D include strains with zoonotic potential involved in extra-intestinal human diseases such as urinary tract infections, neonatal meningitis and septicaemia [44]. Along these lines, the presence of Group B2 strains observed in this study (32.43% of strains fell into this phylogenetic group) is a worrying matter. 

Regarding genes for antibiotic resistance, in the case of *E. coli* and other Gram-negative bacteria, resistance to quinolones is primarily due to mutations in the *gyr*A gene (which codes for the enzyme DNA gyrase). Mutations in the *par*C gene (which codes for the enzyme topoisomerase IV) provide additional resistance [45]. Other resistance mechanisms, such as changes in the permeability of the cell membrane and an increased expression of efflux pumps may also be linked with the acquisition of resistance to these antibiotics. Mutations in the *gyr*A and *par*C genes were detected in all the strains resistant to quinolones and fluoroquinolones in the present research work, a result which agrees with the observations of Gonçalves et al. [46].

Regarding resistance to β-lactams, many bacterial species contain β-lactamases, termed AmpC enzymes and chromosomally encoded by *amp*C genes, which are often detected in strains of intestinal origin [47,48]. Normally these genes are expressed at a low level in *E. coli,* which is insufficient to confer resistance. Nonetheless, a super-expression of the *amp*C sometimes occurs, increasing enzyme activity and generating resistance in the bacteria. This super-expression is generally caused by mutations in *amp*C genes [32]. The 37 strains of *E. coli* tested in this paper contained the *amp*C gene, although a full study of the possible mutations present was not undertaken. 

All the strains with resistance or reduced susceptibility to chloramphenicol harboured the *cml*A gene, which is responsible for the synthesizing of efflux pumps [49]. Other researchers have detected this gene in a high percentage of strains of porcine or avian origin [50,51]. The presence of genes for resistance to chloramphenicol observed in the present work may be surprising given that the use of this antibiotic in animals for human consumption was banned in the 1990s due to its toxicological effects (carcinogenicity and mutagenicity). European Council Regulation 2377/90 laid down a policy of zero tolerance for the presence of chloramphenicol in foodstuffs of animal origin. The three decades that have elapsed since this ban came into force may seem to be long enough for chloramphenicol resistance to disappear. However, cross-resistance and co-resistance mechanisms may have contributed to the persistence over time of genes for resistance to this substance [6,27].

Aminoglycosides inhibit the synthesis of bacterial proteins by binding to ribosomes. Although changes in the permeability of cells and binding points may cause resistance to these antibiotics, the most widespread resistance mechanism is the production of enzymes inactivating these substances [52]. Of the genes determining resistance to aminoglycosides, the *aad*A gene stood out in this work, given that they were detected in over 50% of the strains showing resistance or reduced susceptibility to this family of antibiotics. The values indicated are slightly higher than those reported by other authors [34,53]. The *aac*(3)II and the *aac*(3)IV genes, however, were present in only 10.81% and 5.41%, respectively, of the strains tested in this research. These percentages are lower than seen in other studies of strains of *E. coli* of animal origin [21,46].

None of the strains with resistance or reduced susceptibility to tetracycline tested in this work carried the *tet*A, *tet*B or *tet*C resistance genes. These results do not concur with those of other researchers [9,11,21,34,54], who detected these genes in *E. coli* isolates of animal origin that were resistant to tetracycline. 

Integrons play an important role in increased resistance to antibiotics [55]. These genetic elements hold information for encoding a protein (integrase) that can include or release genes (gene cassettes) such as antibiotic resistance genes (e.g., to sulphonamides). In the present study, the presence of integrons was analysed in all the strains with a phenotype of resistance to sulfamethoxazole-trimethoprim. No strains were noted that had Class 1 (*intI*1) or Class 2 (*intI*2) integrons, and neither was the *qac*EΔ1 gene, usually present in Class 1 integrons, nor the variable region genes *rvintI*1 and *rvintI*2 detected. 

Resistance to sulphonamides is encoded by the *sul*1, *sul*2 and *sul*3 genes, which modify the zones that are targeted by these substances [56]. The importance of the *sul*3 gene should be highlighted due to its considerable capacity to transfer between different populations of bacteria [43]. Noteworthy among the results of the present study was that all the strains with full or partial resistance to sulfamethoxazole-trimethoprim (5.41%) contained the *sul*1, *sul*2 and *sul*3 genes. These results concur with the findings of Radhouani et al. [43], Gonçalves et al. [46] and Momtaz et al. [51] in strains of *E. coli* of animal origin. 

The acquisition of virulence factors, which can occur through horizontal transfer mechanisms, is the outcome of a process of natural selection among the microorganisms present in a host. To this effect, they manage to adapt and survive in the habitat they find themselves in, while simultaneously conferring a pathogenic nature on strains [57]. The emergence of potentially virulent strains that also have a multi-resistant phenotype is an alarming event, which can potentially cause serious clinical problems given the difficulty of treating infections by bacteria with antibiotic resistance [6,43].

Several reports have recorded that strains belonging to phylogenetic Groups B2 and D contain more virulence factors than strains from Groups A and B1 [24]. However, in the present work no differences were noted in the average number of resistance genes or virulence factors among the various phylogenetic groups. 

The *aer* gene makes bacteria able to scavenge iron in conditions where it is scarce, which may happen inside infected animals, increasing bacterial ability to compete for nutrients and, in consequence, bacterial survival [58]. The presence of this gene has been linked to human urinary tract infections, neonatal meningitis and septicaemia [59]. Suárez et al. [60] studied *E. coli* isolates from the intestines of pigs in north-west Spain, finding that 12% of strains carried the *aer* gene. This value is much lower than was observed in the present study, in which the *aer* gene was detected in 56.76% of the strains (69.57% of those in phylogenetic Group B1, 41.67% in Group B2 and 0.00% in Group A). Furthermore, the results obtained from the present research are similar to those reported by Gonçalves et al. [46], who did not find this gene in strains from Group A, but did find it in the majority of strains from Group B1. On the other hand, Radhouani et al. [43] detected this gene in 40% of the strains belonging to Group B2, while it was not present in phylogenetic Groups A and B1.

The *cnf1* gene, which determines the cytotoxic necrotizing factor, was detected in 21.74% of the strains from Group B1 and 25.00% of those from Group B2. These percentages are much higher than those recorded by other researchers, whether the isolates were of clinical origin (3%) [61] or collected in the environment (0% to 5.6%) [43,62].

The *fim*A gene, which encodes the major sub-unit of *E. coli* type 1 fimbriae that enable *E. coli* to adhere to epithelial cells thus encouraging infection, was detected in all the strains in the phylogenetic Groups B1 and B2, but was not present in those from Group A. These results agree with the findings of Radhouani et al. [43], who also observed the presence of this gene in 100% of the strains from Groups B1 and B2. Notably, however, these authors differed from the present study in that they also detected the *fim*A gene in 52.9% of the strains belonging to the phylogenetic Group A. Likewise, Gonçalves et al. [46] found that 80% of the strains in phylogenetic Group A carried this gene.

The *hly* gene, which gives rise to the production of toxins, was found by Tarchouna et al. [61] in 19% of the strains of *E. coli* isolated from patients with urinary tract infections. Interestingly, this figure is lower than the prevalence found in the current study, in which the *hly* gene was detected in all the strains belonging to phylogenetic Group B2 and in 17.39% of the strains from Group B1. For their part, Flores et al. [63] studied strains of *E. coli* isolated from lakes and springs in Portugal and found this gene in just a single strain belonging to Group B2.

The *pap*GIII gene triggers the formation of P-related (Prs) fimbriae, which are actively involved in urinary tract infections. It was present in (50.00%) of the strains in phylogenetic Group A and in 66.67% of the strains from Group B2. Other research carried out on strains of *E. coli* of animal origin found varying but generally slight prevalence, ranging from 0% [43] to 15% [64]. 

The *pap*C gene is present in the strains of *E. coli* responsible for urinary infections. It is essential for the formation of the fimbriae of the pyelonephritis-associated pili (Pap) type, which enable the bacteria to adhere to the epithelium of the urinary tract [65]. This gene was not present in any of the strains trialled. In line with the results reported here, other researchers have found a very low prevalence of the *pap*C gene in bacteria of animal origin [43]. In contrast, when clinical samples from patients with urinary infections were investigated, Tarchouna et al. [61] detected the presence of the *pap*C gene in 41% of the strains of *E. coli* analysed.

Last, the *stx*_1_ gene, which codes for the production of Shiga toxin [1], was not detected in any of the strains studied. This agrees with the findings of other researchers working with isolates of animal origin [62].

## 4. Materials and Methods

### 4.1. Strains

Thirty-seven strains of *Escherichia coli* type I (identified using the miniaturized API 20E system, bioMérieux, Marcy l’Étoile, France) were tested. They were isolated from the surface of 37 pigeon carcasses from north-west Spain, with one isolate studied from each carcass. Twenty-four of the pigeons had been reared in captivity (*Columba livia*) on a diet of hen feed, while 13 were from the wild (*Columba palumbus)*. The domestic pigeons were slaughtered and the wild pigeons were obtained directly from hunters. Immediately after death, the birds were plucked, eviscerated and transported in an ice chest to the laboratory, where they were kept under refrigeration (4 °C). Less than 24 h elapsed from death to analysis. 

### 4.2. Antibiotic Susceptibility Testing

**Figure 3:**
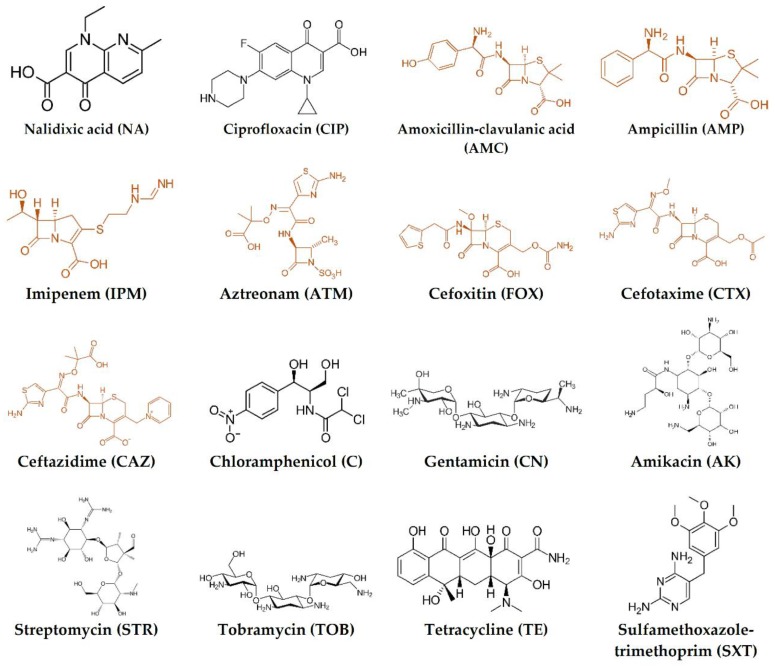
Chemical structure of the 16 antibiotics tested; β-lactams are in red.

The susceptibility of the strains to a total of 16 antimicrobials of clinical importance was tested. The antimicrobials were nalidixic acid (NA; 30 µg), ciprofloxacin (CIP; 5 µg), amoxicillin-clavulanic acid (AMC; 20/10 µg), ampicillin (AMP, 10 µg), imipenem (IPM, 10 µg), aztreonam (ATM, 30 µg), cefoxitin (FOX, 30 µg), cefotaxime (CTX, 30 µg), ceftazidime (CAZ, 10 µg), chloramphenicol (C, 30 µg), gentamicin (CN, 10 µg), amikacin (AK, 30 µg), streptomycin (STR, 10 µg), tobramycin (TOB, 10 µg), tetracycline (TE, 30 µg) and sulfamethoxazole-trimethoprim (SXT, 25 µg) (Figure 3). All the antibiotic discs were obtained from Oxoid Ltd. (Hampshire, England).

The United States National Committee for Clinical and Laboratory Standards (CLSI) guidelines [66] were used to produce the antibiograms (disc diffusion on Mueller-Hinton agar, Oxoid; with incubation for 24 h at 37 °C) and to interpret the inhibition halos. The inhibition zones were measured and scored as sensitive, intermediate (with reduced susceptibility) or resistant. *Escherichia coli* ATCC 25922 and *Staphylococcus aureus* ATCC 29213 were used as the reference strains for the antibiotic disc control.

### 4.3. Phenotypic Determination of Extended Spectrum β-Lactamases (ESBLs)

ESBL phenotypic detection was carried out using the double-disk synergy test. Mueller–Hinton agar plates (Oxoid) were used with discs of the antibiotics ceftazidime, cefotaxime, aztreonam and amoxicillin-clavulanic acid (Oxoid), the latter being placed in the middle of the other three. The discs were placed at a distance of 2 cm from one another and the plates were incubated for 24 h at 37 °C. A phenotype was deemed positive for the production of ESBLs when an increase in the size of the halo was observable in the zone where the inhibition halos of amoxicillin-clavulanic acid intersected with the other three antibiotics. This so-called “ghost zone” is due to the inhibition of ESBLs by clavulanic acid [67].

### 4.4. Genotypic Characterization

For the DNA extraction, the strains were first streaked onto tryptone soya plates (TSA, Oxoid) and incubated for 24 h at 37 °C. They were then transferred to an Eppendorf tube with 0.5 ml of milliQ sterile water and brought to 100 °C in an H-BLOC thermal block (Selecta, Barcelona, Spain) for 8 minutes. Quantification of the extracted DNA was performed with the aid of a NanoDrop 1000 Spectrophotometer (Thermo Fisher Scientific, Inc., West Palm Beach, Florida, U.S.A.). Concentrations of DNA of between 200 ng/μL and 800 ng/μL were considered as acceptable. Once the DNA was extracted, it was stored under refrigeration at 4 ± 1 °C ready for later use.

Polymerase chain reactions (PCRs) were carried out in a total volume of 25 µL made up of 0.5 µM of each primer (Isogen Life Science, Barcelona, Spain), 0.2 mM of deoxynucleoside triphosphates (dNTPs) mix (GeneAmp^®^ dNTP blend, Thermo Fisher Scientific), PCR incomplete buffer 1× (Bioron GmbH, Ludwigshafen, Germany), 1.5 mM of MgCl_2_ (Bioron), 1.25 U of taq DNA polymerase (Bioron) and 5 µl of extracted solution of bacterial DNA. 

DNA amplifications were performed in a Mastercycler (Eppendorf Ibérica S.L.U., Madrid, Spain). The PCR products were separated by horizontal electrophoresis through 1% (wt/vol) agarose gels (Bioron) in TAE buffer 1×, stained with GelRed (Biotium Inc., Hayward, California, U.S.A.), diluted to 1:10,000, and visualized and photographed on a UV transilluminator (Gel Doc™ EZ System; Bio-Rad, Hercules, California, U.S.A.). The size of each PCR product was estimated using standard molecular weight markers (1-kb DNA ladder; Bioron). Negative controls (samples without DNA templates) and positive controls (samples with DNA from positive strains) were included in all PCR assays. 

The phylogenetic group of each strain was determined in accordance with Clermont et al. [68], by means of multiplex polymerase chain reaction (PCR) of the genetic markers *chu*A and *yja*A and the DNA fragment *tsp*E4.C2, using the specific primers and conditions shown in Table 3. The strains were assigned to the phylogenetic groups A (*chua*A- and *tsp*E4.C2-), B1 (*chua*A- and *tsp*E4.C2+), B2 (*chua*A+ and *yja*A+) or D (*chua*A+ and *yja*A-).

Genes for resistance to quinolones (*gyr*A and *par*C), β-lactams (*amp*C), chloramphenicol (*cml*A), streptomycin (*aad*A), gentamicin (*aac*(3)II and *aac*(3)IV), tetracycline (*tet*A, *tet*B and *tet*C), and trimethoprim-sulfamethoxazole (*sul*1, *sul*2 and *sul*3) were studied by specific PCR. The presence of the *intl*1 and *intl*2 genes, encoding Class 1 and 2 integrases, respectively, and their variable region (*vrintl*1 and *vrintl*2) were also analysed by PCR. Additionally, the presence of the *qac*EΔ1 region was studied, given that this is associated with *sul*3 and non-classical Class 1 integrons. The oligonucleotide sequences of the primers used and the amplification conditions are given in Table 4.

The presence of genes involved in the expression of aerolysin (*aer*), necrotizing factor type 1 (*cnf*1), type 1 fimbriae (*fim*A), haemolysin (*hly*), type C fimbriae (*pap*C), adhesin PapG class III (papGIII) and Shiga toxin 1 (*stx*_1_) was also investigated by PCR (Table 5). Positive and negative PCR controls from the bacterial collection of the University of Trás-os-Montes and Alto Douro (Vila Real, Portugal) were used in all the assays.

### 4.5. Statistical Analysis

The prevalence of resistant strains and the prevalence of resistance genes in the various groups of isolates (from both wild and domestic birds) were compared using Fisher’s exact test. Significant differences were set at a probability level of 5% (*P* < 0.05). The Statistica^®^ 8.0 package (Statsoft Ltd., Tulsa, Oklahoma, U.S.A.) was used to carry out the statistical analysis.

## 5. Conclusions

As a general conclusion, it must be highlighted that pigeon meat is a major reservoir of strains of *Escherichia coli* resistant to agents listed as critically important antimicrobials for human medicine, which is of major concern for public health. Moreover, 32.43% of the strains of *E. coli* analysed belonged to phylogenetic groups considered as pathogenic (specifically Group B2) and are normally responsible for extra-intestinal infections in humans. More than 97% of the strains carried one or more virulence factors. These findings are worrying in the context of food safety and public health, not just because of the direct risk of infection through consuming food contaminated with *E. coli*, but also because of the major indirect risk arising from possible horizontal transfers of genes into other pathogenic bacteria. Moreover, the mobility of pigeons, which often make long journeys, facilitates the spread of bacteria carrying resistance genes or virulence factors, or the genes themselves, in the environment. 

The results presented here show that bacteria isolated from birds reared in close proximity to human populations have the highest prevalence of antibiotic resistance. However, resistant bacteria were also found in wild pigeons, indicating that environmental exposure to antimicrobials, antimicrobial residues, resistant bacteria or resistance genes is widespread. To this effect, the presence of strains resistant to antibiotics in ecosystems that have not been subjected to selective pressures enables these animals to be used as biological indicators of resistance to antibiotics. 

## Figures and Tables

**Table 1:** Antibiotic resistance patterns in the thirty-seven *Escherichia coli* strains tested.

Strain Number	Origin	Resistance Pattern
22	Domestic pigeon	AMP/ATM/STR
19	Domestic pigeon	AMP/CAZ/STR
12	Domestic pigeon	AMC/AMP/CAZ/STR
13	Domestic pigeon	AMC/AMP/CAZ/STR
18	Domestic pigeon	AMC/AMP/CAZ/STR
4	Domestic pigeon	AMC/AMP/STR/TE
24	Domestic pigeon	AMP/ATM/ CAZ/CTX
14	Domestic pigeon	AMP/CAZ/CN/STR
16	Domestic pigeon	AMP/CAZ/AK/STR
7	Domestic pigeon	AMP/CAZ/STR/TE
9	Domestic pigeon	AMP/CAZ/STR/TE
1	Domestic pigeon	AMC/AMP/CAZ/STR/TE
5	Domestic pigeon	AMC/AMP/CAZ/STR/TE
6	Domestic pigeon	AMC/AMP/CAZ/STR/TE
10	Domestic pigeon	AMC/AMP/CAZ/STR/TE
11	Domestic pigeon	AMC/AMP/CAZ/STR/TE
21	Domestic pigeon	AMC/AMP/CAZ/STR/TE
23	Domestic pigeon	AMC/AMP/CAZ/STR/TE
15	Domestic pigeon	AMP/CAZ/C/AK/STR
20	Domestic pigeon	AMP/CAZ/AK/STR/TOB
3	Domestic pigeon	AMP/CAZ/CTX/STR/TOB/TE
2	Domestic pigeon	NA/AMC/AMP/CAZ/STR/TOB/TE/SXT
17	Domestic pigeon	CIP/AMC/AMP/CAZ/C/CN/AK/STR
8	Domestic pigeon	AMC/AMP/ FOX/CAZ/C/AK/TOB/TE
28	Wild pigeon	AMP/CAZ
25	Wild pigeon	AMP/CAZ/STR
32	Wild pigeon	AMP/CAZ/STR
37	Wild pigeon	AMP/CAZ/TE
34	Wild pigeon	NA/AMP/CAZ/TE
30	Wild pigeon	AMC/AMP/CAZ/STR
33	Wild pigeon	AMC/AMP/CAZ/STR
27	Wild pigeon	AMC/AMP/CAZ/STR
31	Wild pigeon	AMP/CAZ/STR/TE
36	Wild pigeon	AMP/CAZ/STR/SXT
29	Wild pigeon	AMP/FOX/CAZ/CTX/TE
35	Wild pigeon	AMP/ FOX/CAZ/ STR/TOB/TE
26	Wild pigeon	AMC/AMP/CAZ/C/ CN/STR/TE

Nalidixic acid (NA), ciprofloxacin (CIP), amoxicillin-clavulanic acid (AMC), ampicillin (AMP), aztreonam (ATM), cefoxitin (FOX), ceftazidime (CAZ), cefotaxime (CTX), chloramphenicol (C), gentamicin (CN), amikacin (AK), streptomycin (STR), tobramycin (TOB), tetracycline (TE) and sulfamethoxazole- trimethoprim (SXT).

**Table 2:** Virulence factors present in the *Escherichia coli* strains tested.

Virulence Factors	Number of Strains
*fim*A	4
*pap*GIII	1
*aer/fim*A	14
*cnf1/fim*A	1
*fim*A*/hly*	4
*cnf1/fim*A*/hly*	4
*aer/fim*A*/hly/pap*GIII	5
*cnf1/fim*A*/hly/pap*GIII	1
*aer/cnf1/fim*A*/hly/pap*GIII	2
None	1

**Table 3:** Target genes and primers used in the PCRs performed in this study to determine *Escherichia coli* phylogenetic groups.

Target Gene	Primer Name	Sequence (5′-3′)	Annealing Temperature (°C) (Amplicon Size, bp)	Reference
*chua*A	chuaA-F	GACGAACCAACGGTCAGGAT	55 (279)	[68]
	chuaA-R	TGCCGCCAGTACCAAAGACA		
*tsp*E4.C2	tspE4.C2-F	GAGTAATGTCGGGGCATTCA	55 (152)	[68]
	tspE4.C2-R	CGCGCCAACAAAGTATTACG		
*yja*A	yjaA-F	TGAAGTGTCAGGAGACGCTG	55 (211)	[68]
	yjaA-R	ATGGAGAATGCGTTCCTCAAC		

**Table 4:** Target genes and primers used in the PCRs performed in this study to determine the antibiotic resistance genes and integrons of *Escherichia coli*.

Target Gene	Primer Name	Sequence (5′-3′)	Annealing Temperature (°C) (Amplicon Size, bp)	Reference
*gyr*A	gyrA-F	TACACCGGTCAACATTGAGG	64 (648)	[69]
	gyrA-R	TTAATGATTGCCGCCGTCGG		
*par*C	parC-F	AAACCTGTTCAGCGCCGCATT	55 (395)	[70]
	parC-R	GTGGTGCCGTTAAGCAAA		
*amp*C	ampC-F	AATGGGTTTTCTACGGTCTG	57 (1800)	[71]
	ampC-R	GGGCAGCAAATGTGGAGCAA		
*cml*A	cmlA-F	TGTCATTTACGGCATACTCG	55 (455)	[72]
	cmlA-R	ATCAGGCATCCCATTCCCAT		
*aad*A	aadA-F	GCAGCGCAATGACATTCTTG	60 (282)	[73]
	aadA-R	ATCCTTCGGCGCGATTTTG		
*aac*(3)II	aac(3)II-F	ACTGTGATGGGATACGCGTC	60 (237)	[72]
	aac(3)II-R	CTCCGTCAGCGTTTCAGCTA		
*aac*(3)IV	aac(3)IV-F	CTTCAGGATGGCAAGTTGGT	60 (286)	[72]
	aac(3)IV-R	TCATCTCGTTCTCCGCTCAT		
*tet*A	tetA-F	GTAATTCTGAGCACTGTCGC	62 (937)	[74]
	tetA-R	CTGCCTGGACAACATTGCTT		
*tet*B	tetB-F	CTCAGTATTCCAAGCCTTTG	57 (416)	[74]
	tetB-R	CTAAGCACTTGTCTCCTGTT		
*tet*C	tetC-F	TCTAACAATGCGCTCATCGT	62 (570)	[74]
	tetC-R	GGTTGAAGGCTCTCAAGGGC		
*sul*1	sul1-F	TGGTGACGGTGTTCGGCATTC	63 (789)	[75]
	sul1-R	GCGAGGGTTTCCGAGAAGGTG		
*sul*2	sul2-F	CGGCATCGTCAACATAACC	50 (722)	[76]
	sul2-R	GTGTGCGGATGAAGTCAG		
*sul*3	sul3-F	CATTCTAGAAAACAGTCGTAGTTCG	51 (990)	[56]
	sul3-R	CATCTGCAGCTAACCTAGGGCTTTGGA		
*intl*1	intl1-F	GGGTCAAGGATCTGGATTTCG	62 (483)	[75]
	intl1-R	ACATGGGTGTAAATCATCGTC		
*rvintl*1	rvintl1-F	GGCATCCAAGCAGCAAG	55 (variable)	[77]
	rvintl1-R	AAGCAGACTTGACCTGA		
*qac*EΔ1	qacEΔ1-F	GGCTGGCTTTTTCTTGTTATCG	60 (287)	[75]
	qacEΔ1-R	TGAGCCCCATACCTACAAAGC		
*intl*2	intl2-F	CACGGATATGCGACAAAAAGGT	62 (788)	[75]
	intl2-R	GTAGCAAACGAGTGACGAAATG		
*rvintl*2	rvintl2-F	CGGGATCCCGGACGGCATGCACGATTTGTA	60 (variable)	[78]
	rvintl2-R	GATGCCATCGCAAGTACGAG		

**Table 5:** Target genes and primers used in the PCRs performed in this study to determine the *Escherichia coli* virulence factors.

Target Gene	Primer Name	Sequence (5′-3′)	Annealing Temperature (°C) (Amplicon Size, bp)	Reference
*aer*	aer-F	TACCGGATTGTCATATGCAGACCGT	63 (602)	[79]
	aer-R	AATATCTTCCTCCAGTCCGGAGAAG		
*cnf*1	cnf1-F	AAGATGGAGTTTCCTATGCAGGAG	63 (498)	[79]
	cnf1-R	CATTCAGAGTCCTGCCCTCATTATT		
*fim*A	fimA-F	GTTGTTCTGTCGGCTCTGTC	55 (447)	[80]
	fimA-R	ATGGTGTTGGTTCCGTTATTC		
*hly*	hly-F	AACAAGGATAAGCACTGTTCTGGCT	63 (1177)	[79]
	hly-R	ACCATATAAGCGGTCATTCCCGTCA		
*pap*C	papC-F	GACGGCTGTACTGCAGGGTGTGGCG	63 (328)	[79]
	papC-R	ATATCCTTTCTGCAGGGATGCAATA		
*pap*GIII	papGIII-F	CATTTATCGTCCTCAACTTAG	55 (482)	[80]
	papGIII-R	AAGAAGGGATTTTGTAGCGTC		
*stx*_1_	stx_1_-R	ATAAATCGCCATTCGTTGACTAC	65-60 (180)	[81]
	stx_1_-R	AGAACGCCCACTGAGATCATC		
